# Corrosion and Thermal Stability of CrMnFeNi High Entropy Alloy in Molten FLiBe Salt

**DOI:** 10.1038/s41598-019-55653-2

**Published:** 2019-12-12

**Authors:** Mohamed Elbakhshwan, William Doniger, Cody Falconer, Michael Moorehead, Calvin Parkin, Chuan Zhang, Kumar Sridharan, Adrien Couet

**Affiliations:** 1 Engineering Physics Department, University of Wisconsin Madison, Madison, WI, USA; 2 Material Science and Engineering Department, University of Wisconsin Madison, Madison, WI, USA; 3 Computherm Inc, Middleton, WI, USA

**Keywords:** Structural materials, Metals and alloys

## Abstract

The corrosion behavior of the FCC Cr_18_Mn_27_Fe_27.5_Ni_27.5_ high entropy alloy (HEA) after exposure to molten FLiBe salt at 700 °C for 1000 hours, has been investigated. Results show that the HEA lost a higher mass compared to the reference 316 H stainless steel due to the dissolution of Mn into the molten salt. The loss of Mn from the alloy appeared to discourage the dissolution of Cr in the molten fluoride salts which is widely recognized as the mechanism of corrosion degradation. Thermal exposure at 700 °C for 1000 hours also led to the precipitation of an additional BCC phase Cr_67_Fe_13_Mn_18.5_Ni_1.5_, which was confirmed by CALPHAD predictions.

## Introduction

Corrosion resistance of alloys in light water reactors (LWRs) and more generally in oxidative environments is achieved by the formation of protective oxide layers that hinder further oxidation. Alloys containing Cr, Al, or Si typically tend to form a compact and self-healing chromia (Cr_2_O_3_), alumina (Al_2_O_3_) or silica (SiO_2_) layers, respectively, which acts as effective diffusion barriers against further oxidation^1–3^. However, in molten fluoride salts (salt of choice for many molten salt reactor concepts, MSRs) these protective oxide layers on structural alloys are unstable which in turn leads to the dissolution of the least noble element in the alloy. Such a corrosion mechanism can lead to a reduction in components’ wall thickness and eventually the loss of the structural integrity of the reactor components^1,2^. The dissolution products themselves may lead to plate-out in the relatively cooler sections of the system due to the strong sensitivity of solubility of analytes to temperature.

Development of materials suitable for molten fluoride salt environments started in the 1950s with the Aircraft Reactor Experiment (ARE)^4^. This experiment used Inconel 600 as a container material, however it was found that this material was not be suitable for high temperature molten fluoride salt applications. The Molten Salt Reactor Experiment (MSRE) at ORNL led to the development of Hastelloy N alloy as a structural material for fuel-bearing molten fluoride salt, which was demonstrated to have superior long-term corrosion resistance in molten fluoride salts. However, in fuel bearing molten salts, the fission product tellurium was noted to lead to crack formation in the alloy due to the precipitation of intermetallic compounds at the grain boundaries^2^. In addition, in such high Ni-content alloys, additional embrittlement may be caused during irradiation. Transmutation of Ni can lead to the formation of helium that weaken the alloy grain boundaries^2^. Several other traditional high temperature alloys have been tested in molten fluoride salts such as 316 L and 304 stainless steels, Incoloy 800 H, Inconel 617, Inconel 625, Hastelloy X, and Haynes 230^5–8^.

This study focuses on the corrosion behavior of a new class of alloys in molten salts, namely High Entropy Alloys (HEAs) which are composed of four or more elements mixed in equimolar or near to equimolar ratio as a single phase solid solution^9^. Since their inception^10,11^, HEAs’ development has gained considerable attention due to their high thermal stability, good mechanical properties, and high wear resistance^10–13^. Additionally, some HEAs have shown high corrosion resistance in aqueous solutions comparable to austenitic and ferritic stainless steels^14^. HEAs are generally characterized by three main phenomena: (1) high entropy of mixing due to the different principal constitutive elements, (2) high lattice distortion due to the various atomic sizes in the unit cell, and (3) sluggish diffusion of defects in HEAs^13^. However, the third hypothesis is not well supported by experimental data and only few studies have shown signs of slowing down of species diffusion in HEAs^13^.

For in-core nuclear reactor applications, certain HEAs have shown high radiation damage tolerance especially at high dpa (displacements per atom) levels. This is speculated to be due to the above-mentioned properties which promote the vacancy-interstitial recombination rate under irradiation^15–17^. For example, the radiation stability of Cr_18_Fe_27_Mn_27_Ni_27_ HEA has been studied by ion irradiation of Ni ions at energies of 3 and 5.8 MeV at room temperature and up to 700 °C and damage levels range from 0.1 to 10 dpa^17^. The alloy showed excellent radiation damage resistance, compared to conventional austenitic Cr-Fe-Ni and Cr-Mn-Fe alloys, in terms of suppression of radiation-induced solute segregation, high swelling resistance, and lower defect cluster size with higher number density. Additionally, no phase separation was observed under the studied conditions. Such properties make this alloy attractive for in-core nuclear reactor applications and more specifically in MSRs if corrosion resistance can be demonstrated.

This study aims to test the effect of the elemental stability inherent in HEAs due to the high configurational entropy and/or sluggish diffusion (if any) on the elemental dissolution in the molten salt. Specifically, the study focuses on corrosion performance and thermal stability of the Cr_18_Mn_27_Fe_27.5_Ni_27.5_ alloy in molten fluoride salt environment. This multi-component alloy with both Mn and Cr which are reactive in molten fluoride salt is well suited to test these hypotheses. This high entropy alloy was exposed to molten FLiBe salt at 700 °C for 1000 hours followed by detailed analysis of corrosion in the near-surface regions of the alloy in terms of elemental dissolution into the molten salt and morphological changes at the surface. The results were compared to that of 316 H stainless steel tested under similar conditions.

## Results

### Pre-corrosion examinations

**Figure 1:**
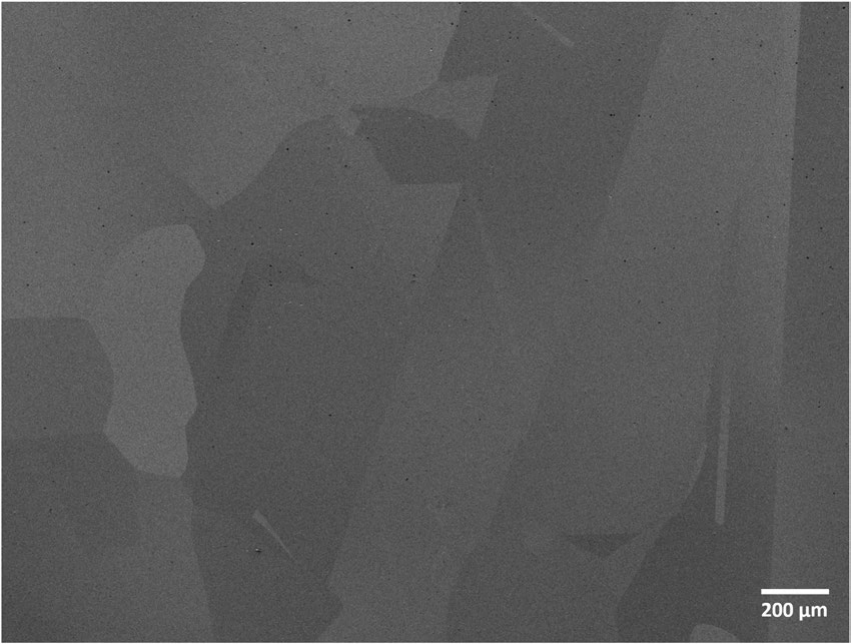
SEM micrograph of Cr_18_Mn_27_Fe_27.5_Ni_27.5_ HEA produced by arc-melting and then homogenized at 1200 °C for 48 hours followed by cooling in an inert environment.

**Figure 2:**
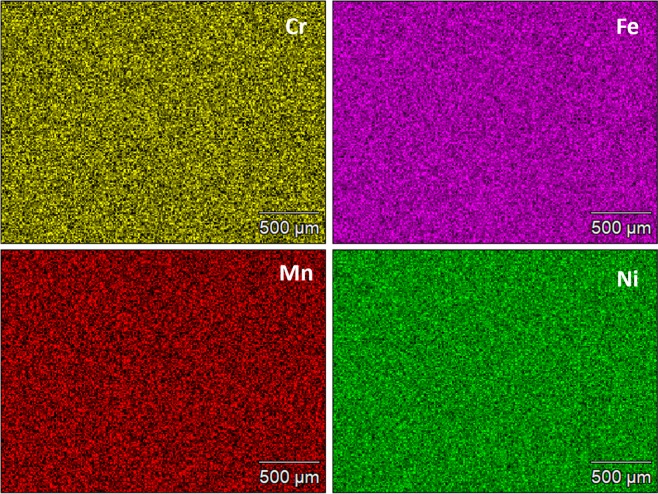
Elemental mapping of elements in the Cr_18_Mn_27_Fe_27.5_Ni_27.5_ HEA after homogenization at 1200 °C for 48 hours followed by cooling in an inert environment.

**Figure 3:**
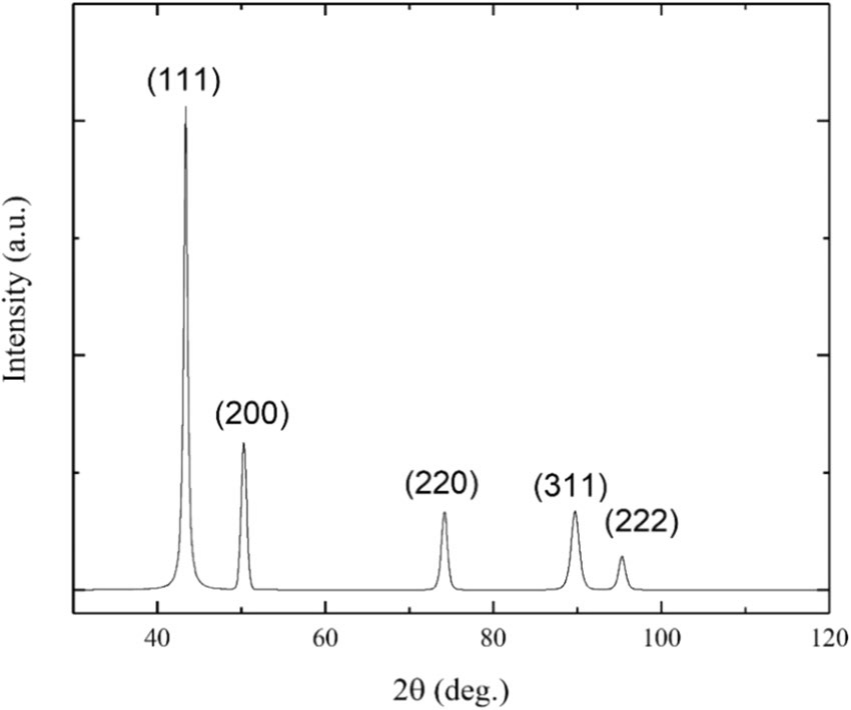
X-ray diffraction for homogenized Cr_18_Mn_27_Fe_27.5_Ni_27.5_ HEA demonstrating it to be a fully FCC solid solution, devoid of any second phases.

The microstructure of the Cr_18_Mn_27_Fe_27.5_Ni_27.5_ alloy prepared for this work is shown in Fig. 1. The grain size is quite large, on the order of hundreds of micrometers and elemental EDS mapping revealed that the material is compositionally homogeneous and devoid of segregation as shown in Fig. 2. Limited porosity was observed in the microstructure, possibly due to oxygen impurities from the precursor material, solidification shrinkage, or Kirkendall voiding during homogenization treatment^18^. The XRD pattern shown in Fig. 3 confirmed the alloy to be single phase, polycrystalline material with FCC crystal structure.

### Post-corrosion examinations

**Figure 4:**
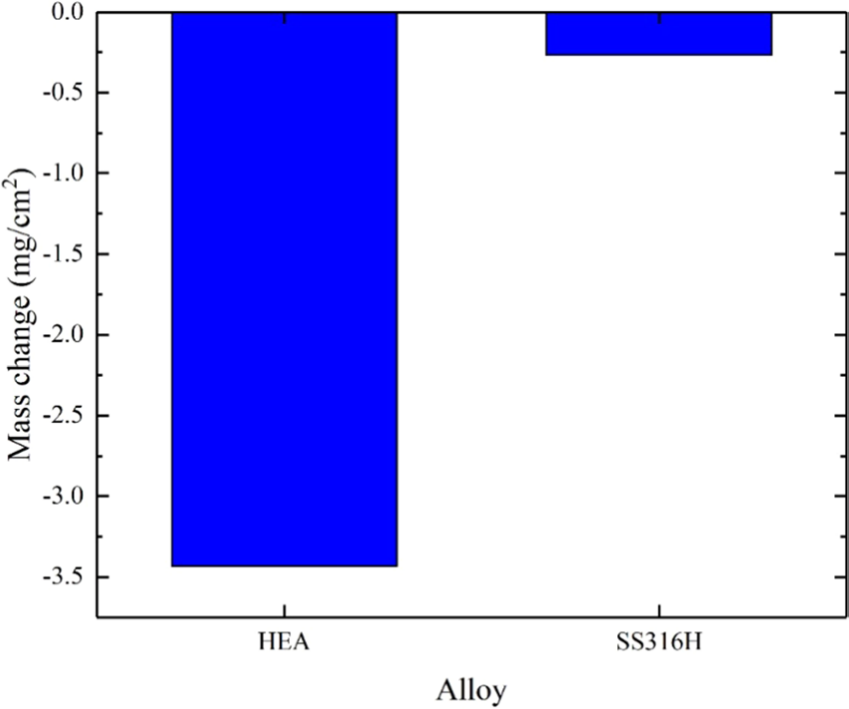
Mass change for Cr_18_Mn_27_Fe_27.5_Ni_27.5_ and 316 H stainless steel alloys after exposure to molten FLiBe salt at 700 °C for 1000 hours.

Mass change per unit area of the FCC HEA was compared to that of 316 H stainless steel as a reference material tested under identical conditions, and the results are shown in Fig. 4. The results show that the HEA experienced a significantly higher mass loss due to elemental dissolution in the FLiBe salt, of about 3.4 mg/cm^2^, compared to 0.26 mg/cm^2^ mass loss in the reference 316 H stainless steel. This large difference in mass loss indicates that elemental dissolution occurred more rapidly in the HEA than 316 H stainless steel.

**Figure 5:**
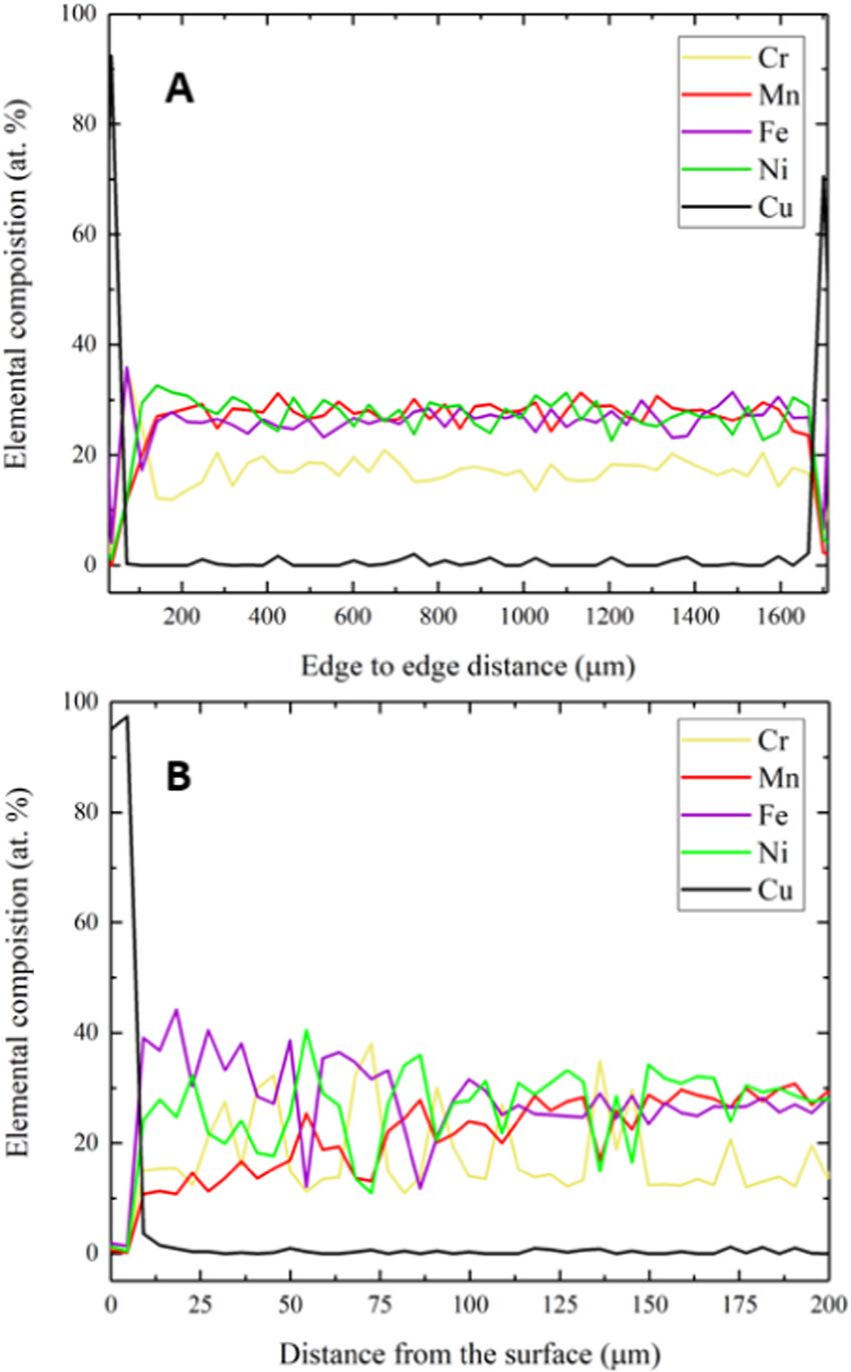
EDS scan for Cr_18_Mn_27_Fe_27.5_Ni_27.5_ sample after exposure to molten FLiBe salt at 700 °C for 1000 hours. (**A**) edge to edge scan and (**B**) near-surface scan. Cu was used as a protective coating for surface during cross-sectional sample preparation.

**Figure 6:**
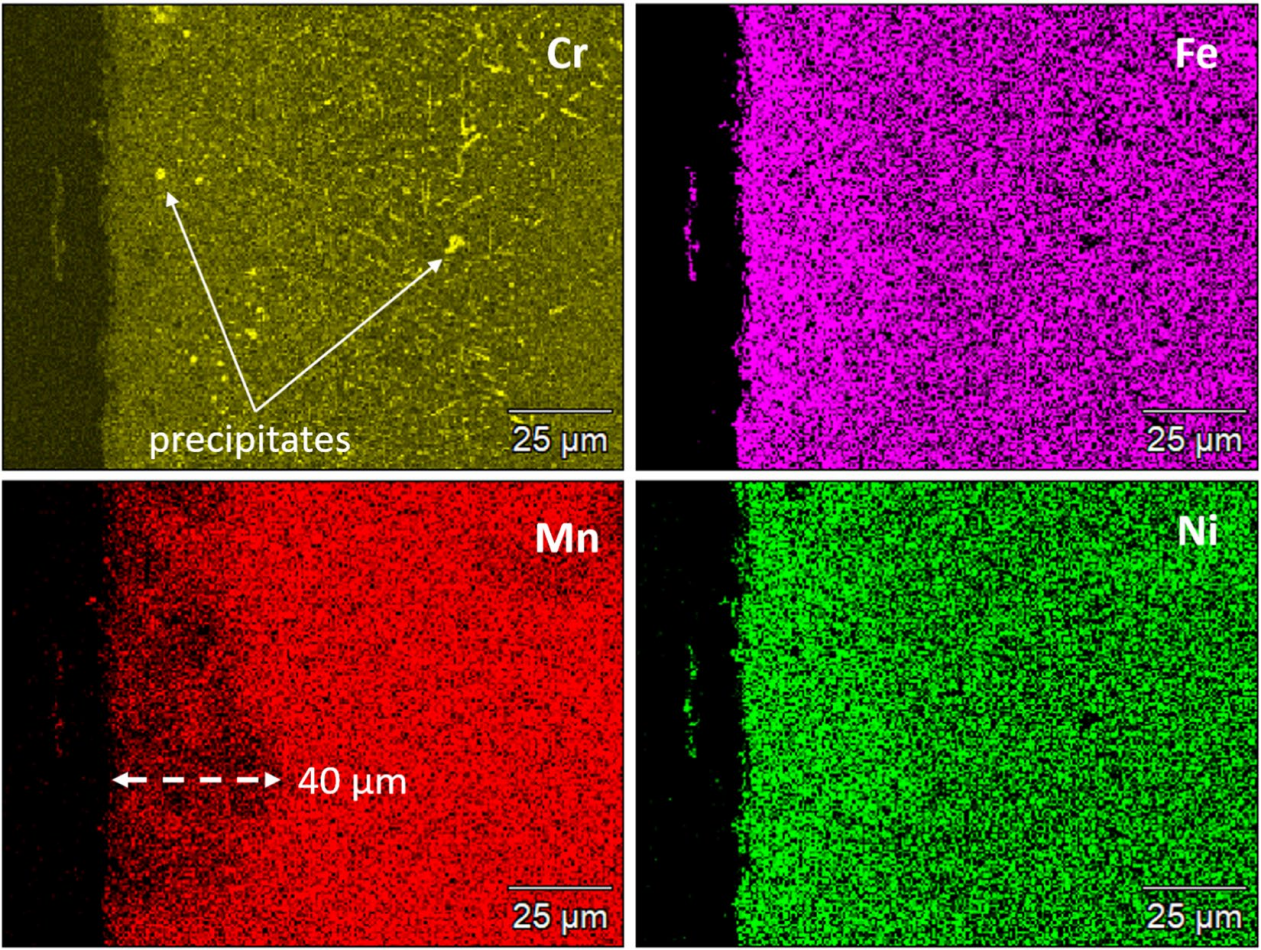
Cross-sectional SEM-EDS elemental distribution x-ray maps of in the near-surface regions of Cr_18_Mn_27_Fe_27.5_Ni_27.5_ HEA after exposure to molten FLiBe salt at 700 °C for 1000 hours. Results show uniform distrubition of Ni and Fe. Mn shows significant depeletion in the near-surface region. Cr-rich particles were precipitated as indicated by the white arrows.

**Figure 7:**
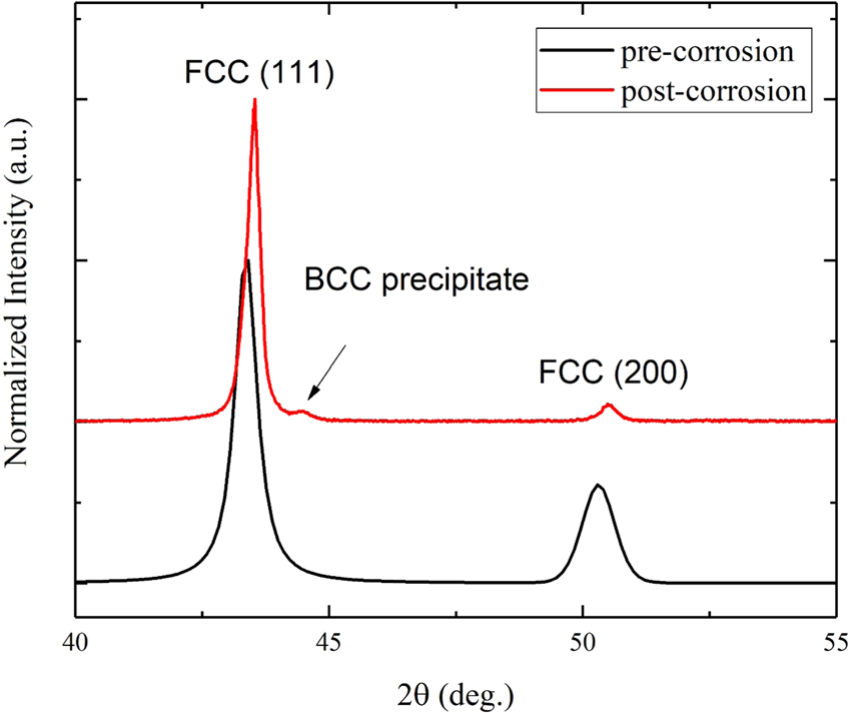
Bulk X-ray diffraction for homogenized Cr_18_Mn_27_Fe_27.5_Ni_27.5_ alloy pre- and post-corrosion in molten FLiBe salt at 700 °C for 1000 hours. In the pre-corrosion condition, the alloy was found to be a fully FCC solid solution, while the long-term annealing led to the formation of islands of BCC precipitates.

To evaluate the depth of attack, EDS line scans on the sample cross-section were performed. Edge to edge scan showed that all elements maintain the nominal alloy composition throughout the thickness of the sample, except for Mn which shows a depletion in the near-surface regions at both edges as shown in Fig. 5A. Figure 5B shows a higher resolution scan close to the sample surface. The line scan confirms Mn depletion that extends to over 100 µm from the surface and shows discrete spots with noticeably higher Cr concentration. Elemental mapping in Fig. 6 shows that Mn depletion in the near-surface region extended up to a depth of approximately 40 µm. Needle-shaped Cr-rich particles were found to precipitate and bulk XRD patterns showed that a new precipitate phase was formed after the corrosion/annealing process as shown in Fig. 7.

The chemical analysis of the FLiBe salt before and after corrosion of the HEA and 316 H stainless steel alloys were performed and are summarized in Table 1. For the salt exposed to the 316 H stainless steel, the Cr concentration increased from 6 to 263 ppm while there was negligible change in the Cr concentration of the salt exposed to the HEA alloy after corrosion tests. The Mn concentration showed interesting behavior, it increased from 4 to 349 ppm in the salt exposed to the HEA alloy, and also increased in salt exposed to the 316 H stainless steel alloy from 4 to 40 ppm. This significant change indicates the higher rates of Mn dissolution in the salt, while Ni experienced slight reduction in both cases. The diffusion of Mn is likely occur via vacancy diffusion^19,20^, but no significant change in the porosity concentration was observed after the corrosion test.

**Table 1:** Trace elemental analysis in the FLiBe salt before and after corrosion tests, performed using inductively coupled plasma-optical emission spectroscopy (ICP-OES)^19^.

Element	Pre-corrosion (ppm)	Post-corrosion of the HEA alloy (ppm)	Post-corrosion of 316 H SS alloy (ppm)
Chromium	6 ± 1.3	10 ± 0.3	263 ± 7.8
Nickel	14 ± 2.7	8 ± 0.3	8 ± 0.3
Manganese	4 ± 0.4	349 ± 5.3	40 ± 1.1
Iron*	ND	ND	ND

*ND: Non detectable.

## Discussion

### Corrosion behavior

Corrosion in molten salts is affected by several factors such as thermodynamics of corrosion reactions, elemental activities in the salt and the alloy, impurities, and temperature gradients. For instance, moisture in the salt can lead to the formation of hydrofluoric acid which can interact with metallic structural alloys and cause elemental dissolution into the salt. Also, metallic impurities incorporated in the salt may undergo exchange reactions to leach the least noble element out of the alloy^2^. Most engineering alloys used in nuclear applications contain reasonable amounts of Cr to provide protection against air oxidation, and typically, chromium is the most vulnerable alloying element to fluoride salt corrosion^2^.

**Figure 8:**
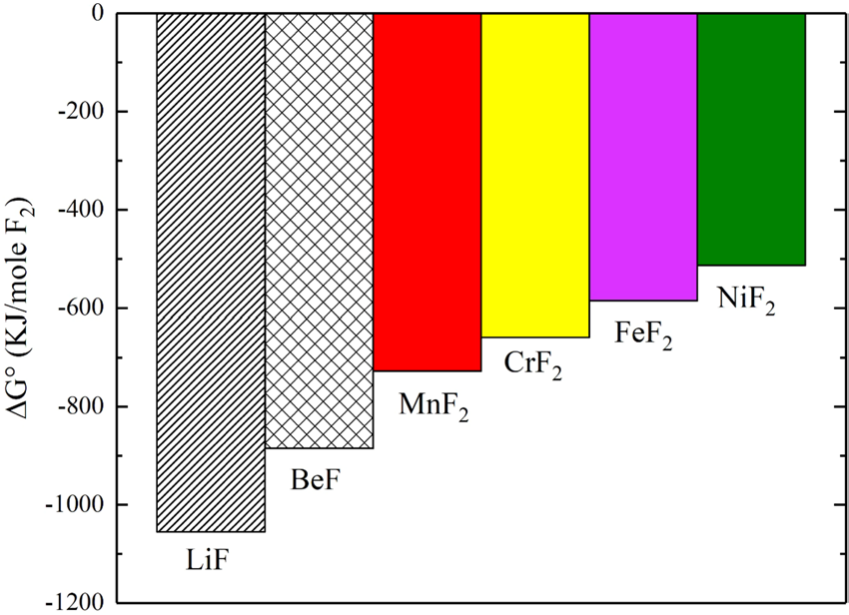
Standard Gibbs free energy of formation for the various fluoride compounds of relevance to this study at 700 °C^21^.

The trace elemental analysis (Table 1) showed that, although both HEA and 316 H stainless steel alloys have almost the same Cr concentration (~18 wt.%), the HEA alloy lost significantly lower amount of Cr to the salt compared to the 316 H stainless steel. This is due to the presence of Mn in the HEA that hinders the dissolution of Cr in the salt. Even for the 316 H stainless steel which contains an appreciably lower Mn concentration (maximum 2 wt.%), Mn dissolution was observed raising its concentration in the salt from 4 to 40 ppm after the corrosion test. These results may be explained by the lower standard Gibbs free energy of formation of MnF_2_ compared to CrF_2_, as shown in Fig. 8, which suggests that Mn would tend to dissolve in molten fluoride salt rather than Cr^21^. In addition, the mass change measurements (Fig. 4) showed that the HEA has a higher mass loss compared to the 316 H stainless steel alloy, which indicates a higher dissolution rate of Mn compared to Cr, likely due to the higher diffusion coefficient of Mn in these systems^22,23^. The combination of lower standard Gibbs free energy and high diffusion coefficient of Mn relative to Cr, led to the higher dissolution rate of Mn in the salt that hinder the dissolution of Cr.

The dissolution of Mn is governed by the presence of the more noble metallic impurities in the molten salt (Eqs. 1–3) and the interaction with the fluorine compounds, which is dictated by the fluorine partial pressure in the salt (Eqs. 4 and 5). Indeed, the original fluorine potential may be relatively high in the salt (i.e. larger than the Mn/MnF_2_ equilibrium potential), considering the HF/H_2_ sparging process in a Ni vessel was used for salt purification. The following equations summarize the possible Mn dissolution mechanisms as follow:

1 $${{\rm{NiF}}}_{2}+{\rm{Mn}}={{\rm{MnF}}}_{2}+{\rm{Ni}}$$

2 $${{\rm{FeF}}}_{2}+{\rm{Mn}}={{\rm{MnF}}}_{2}+{\rm{Fe}}$$

3 $${{\rm{CrF}}}_{2}+{\rm{Mn}}={{\rm{MnF}}}_{2}+{\rm{Cr}}$$

4 $$2{\rm{HF}}({\rm{g}})+{\rm{Mn}}={{\rm{MnF}}}_{2}+{{\rm{H}}}_{2}$$

5 $${{\rm{F}}}_{2}({\rm{g}})+{\rm{Mn}}={{\rm{MnF}}}_{2}$$

These chemical reactions suggest that the presence/dissolution of Mn hindered the dissolution of Cr in the molten fluoride salt, leaving behind the alloy surface with almost the same concentration of Cr as in the bulk alloy as shown in Fig. 5. These findings are in contradiction to most engineering alloys investigated in literature, where Mn concentration is low or non-existent and Cr is considered the most active element and tends to leach out upon exposure to molten fluoride salt.

The results of the present study show that Cr did not dissolve from the stainless steel-like HEA into the salt although the electronegativity difference between fluorine and chromium is quite significant. This indicates that Mn may act as a sacrificial element to protect Cr from dissolution in molten fluoride salts since it has lower free energy of formation as shown in Fig. 8. Two mechanisms could be at play here. Following the significant Mn dissolution from the alloy into the salt to form MnF_2_, the Mn/MnF_2_ redox couple could become a salt redox buffer, and thus the immersed sample, would then be fixed at this buffer level. At this potential, the Cr becomes a noble element with respect to the salt, and Cr dissolution would not be thermodynamically favorable. It is not known how much Mn in the salt is required to reach a satisfactory buffer level, but that would be an interesting point to study in the future. These observations point to the possibility of using Mn/MnF_2_ couple as a redox buffer to reduce the overall corrosion potential and to slow or stop the dissolution of Cr. Such technique, in principle, was employed in the MSR experiment in ORNL during the 1960s by adding metallic elements such as Mg, Zr, and Be to the salt^2,24–26^. However, in our case, the structural materials would provide a constant source of buffer to the salt. Another possible mechanism is that adding Mn to the alloy in molten salt environment could act as a sacrificial mechanism to protect the Cr content. This is relatively different from aqueous sacrificial protection mechanism, where Mn would be added as a pure Mn bar in electrical contact with the alloy of interest rather than as an alloying element. Indeed, sacrificial alloying is not a successful corrosion protection method in aqueous systems due to the limited diffusivity of elements at low temperatures^27^. Thus, even if the first monolayers of Mn atoms react preferentially with the salt compared to Cr atoms, Mn diffusivity to the surface would be too slow to prevent Cr from reacting. However, at high temperatures such as 700 °C, the test temperature in our study, Mn diffusivity is significantly increased such that a steady supply of Mn to the salt-alloy interface is more likely to prevent significant amount of Cr atoms from reacting with the salt. The two mechanisms outlined above could both play a role in preventing Cr depletion from the stainless steel-like HEA.

It is worth mentioning that, most engineering alloys have certain amounts of Mn as a minor alloying element including Hastelloy N (max. 0.8 wt.%) which is optimized for molten salt applications. This means that consideration must be given to the Mn concentration and similar minor alloying elements with high formation energy of fluorides because they will tend to dissolve in the salt faster than the principle elements which may affect the salt chemistry and the redox potential.

Meanwhile, the corrosion behavior of the Cr_18_Mn_27_Fe_27.5_Ni_27.5_ high entropy alloy seems to follow mechanisms observed in conventional alloys, which is controlled by the thermodynamic stability of corrosion products relative to fluorine potential and the level of impurities in the salt. Similar results were observed during the corrosion of CrFeNiCo, CrFeNiCoMn, and CrFeNiCuMn high entropy systems in various aqueous solutions^28–31^. That being said, it is not sufficient to draw conclusions about the role of the high entropy of mixing and sluggish diffusion in the corrosion behavior of HEAs. More experimental results with various HEAs in different salt media would be needed to better understand the behavior of HEAs in these corrosive environments.

### Thermal stability and applicability to nuclear applications

**Figure 9:**
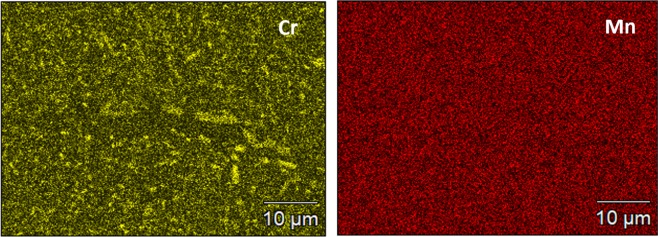
Bulk elemental distribution in the Cr_18_Mn_27_Fe_27.5_Ni_27.5_ alloy after exposure to molten FLiBe salt at 700 °C for 1000 hours. Results show wide distrubition of thermally-induced Cr-rich precipitate particles in the entire thickness of the sample.

The use of HEAs in nuclear applications depends greatly on their microstructural stability in the extreme environments of the operating conditions. Although Cr_18_Mn_27_Fe_27.5_Ni_27.5_ alloy shows good mechanical properties and relatively high resistance to radiation damage^17^, this study showed that the alloy experienced phase instability at 700 °C after 1000 hours. Cr-rich particles were found to precipitate in the alloy, even well beneath the corrosion affected region, after the high temperature exposure during the corrosion test. Just underneath the surface, few fine circular Cr-rich precipitates were observed. As the distance from the surface increased, the Cr-rich precipitates’ concentration increased and their morphology became more needle-shaped as shown in Fig. 6. Then, the precipitates’ concentration decreased significantly but persisted through the entire thickness of the sample as shown in Figs. 6 and 9. These changes in the concentration and morphology of the Cr-rich precipitates indicate that the precipitation process not only depends on both the annealing temperature and duration, but also on the Mn concentration in the alloy, which varies due to its dissolution in the salt.

Several microstructural changes were also observed in the bulk X-ray diffraction analysis as shown in Fig. 7. First, all the FCC peaks exhibited a shift toward higher 2*θ* values (smaller lattice parameter) which indicates the bulk lattice contracts due the formation of new precipitates and long-term annealing. Secondly the ratio between the FCC (200) and the (111) reflections (I_200_/I_111_) decreased after the annealing process due to (111) grain growth. Finally, the newly formed precipitates exhibited an ordered phase with a lower lattice parameter than the original FCC phase.

**Figure 10:**
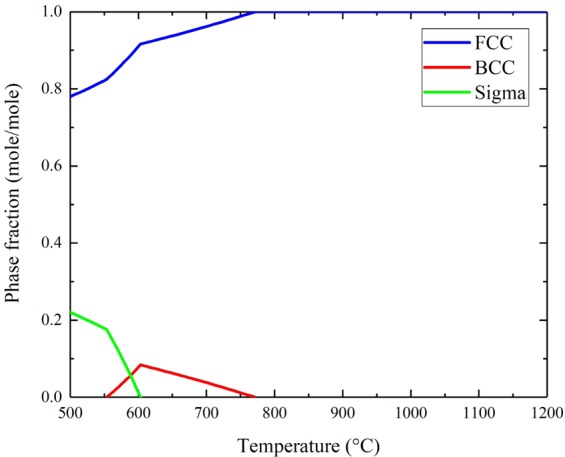
Phase diagram of Cr_18_Mn_27_Fe_27.5_Ni_27.5_ alloy from 600 °C to 1200 °C predicted using CAPHAD showing co-existence of the FCC and BCC phase at 700 °C.

**Figure 11:**
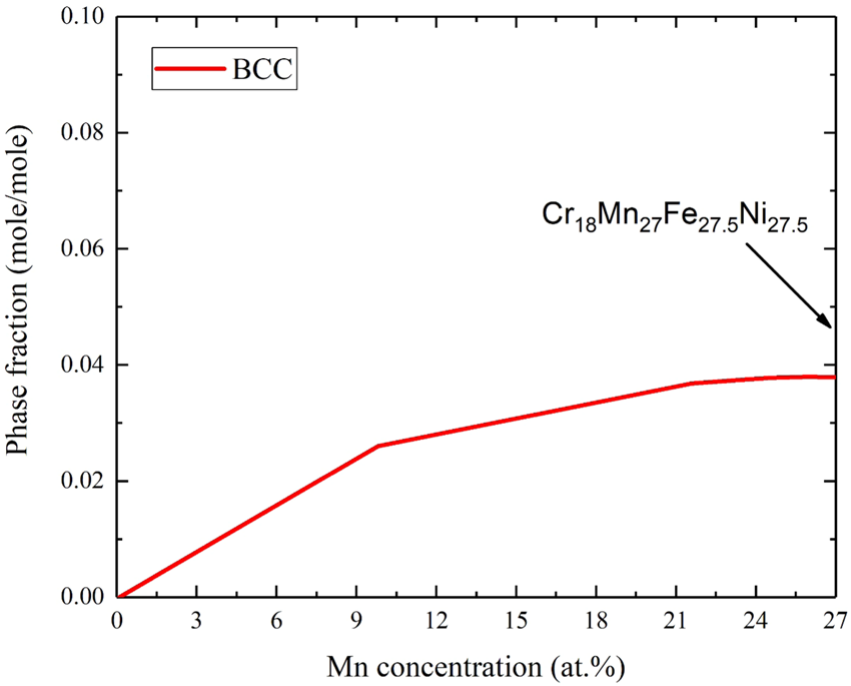
BCC precipitate phase fraction in the Cr_18_Mn_27_Fe_27.5_Ni_27.5_ alloy as a function Mn concentration.

In order to confirm the above findings, CALPHAD calculations were performed to determine all the equilibrium phases for the Cr_18_Mn_27_Fe_27.5_Ni_27.5_ alloy. Results show that the alloy is not expected to maintain the single phase structure in temperatures ranging from 500 °C up to 770 °C as shown in Fig. 10. At 700 °C, the alloy is expected to contain both FCC and BCC phases. The BCC phase is approximately 4% in phase fraction and is rich in Cr and low in Ni with nominal composition of Cr_67_Fe_13_Mn_18.5_Ni_1.5_. In addition, CALPHAD calculations were used to evaluate the relationship between the Mn dissolution and the concentration of precipitates. At 700 °C, as Mn diffuses out of the Cr_18_Mn_27_Fe_27.5_Ni_27.5_ alloy, the BCC phase fraction would decrease from approximately 3.7 at.% in the original alloy to 2.8 at.% when the Mn concentration reduced to 12 at.%, and then to 0.1 at.% after complete depletion of Mn as shown in Fig. 11. These results would explain the relatively low concentration of Cr-rich precipitate in the Mn-depleted zone shown in Fig. 6.

The agreement between our experimental results (Figs. 6 and 7) and the CALPHAD calculations (Fig. 9) confirms that the Cr_18_Mn_27_Fe_27.5_Ni_27.5_ alloy does not form a stable solid solution at 700 °C, which stresses the need for long term aging experiments to assess HEA phase stability. Although this alloy has been studied under various conditions of high temperatures and radiation damage levels, no study has reported any phase separation^17,32,33^. All these studies reported the microstructure after the homogenization process at high temperature (1200 °C), which is shown to induce only a single phase solid solutions as shown in Fig. 9. On the other hand, all the irradiation and mechanical testing were performed at lower temperatures where phase separation is expected to occur, but experimental tests have to be performed for a long enough time for phase separation to occur. These results show the importance of the thermodynamic calculations in predicting the phase stability in HEAs and informing further decisions on alloy selection.

Although it was not reported in the literature that this alloy would form second phase precipitates^17^, our experimental and computational results show that such precipitation occurs. At these levels, this precipitation could be beneficial to nuclear applications. Indeed, second phase precipitates may increase the high-temperature strength of the alloy. This is important since presently, the only alloy with demonstrable data as structural material for MSRs is Hastelloy-N, but it does not have sufficient creep strength^2^. In addition, precipitate/matrix interfaces can act as irradiation induced point defect sinks, increasing the alloy’s resistance to irradiation damage. Finally, since Cr is immobilized as precipitates in the surface regions beneath the alloy/salt interface, it could not potentially dissolve in the salt and would still be available at levels sufficient to prevent air oxidation on the outer side of the piping. Thus, both the molten salt (depletion of Mn) and ageing (Cr rich precipitate) are effects occurring in-operando which could improve the resistance of this HEA to degradation in MSR environment.

The nuclear industry is continually looking for new materials with enhanced radiation and corrosion resistance and HEAs could provide new approaches to design and formulation of alloy compositions that meet and exceed those requirements. Optimizing alloy compositions, alloying elements, and precipitation could provide useful directions to the development of alloys with superiors performance in such challenging environments.

## Experimental Methods

50 gram ingots of Cr_18_Mn_27_Fe_27.5_Ni_27.5_ alloy were prepared by arc melting, using an Arcast Arc 200 unit. The alloys produced using this method typically have a dendritic structure often with pronounced solute segregation. High-temperature homogenization was performed at 1200 °C in a vacuum environment for 48 hours in quartz tubes with a titanium getter to sequester any residual oxygen. After the homogenization step, the quartz tubes were air-cooled to room temperature and the resulting solidified rod was sectioned into uniform discs.

Samples were then exposed to a molten salt mixture of 2LiF-BeF_2_, commonly referred to as FLiBe, at a temperature of 700 °C for 1000 hours. FLiBe salt was produced and purified at UW-Madison. Lithium fluoride (99.8% purity) was obtained from Noah Technologies and beryllium fluoride (98.8% purity) was purchased from Materion. A 40 kg batch was purified by hydro-fluorination, a process in which a hydrogen and gaseous hydrofluoric acid mixture are sparged through the salt melt. This process removes sulfur, oxygen, and moisture as well as some metallic impurities in the salts such as Fe and Ni^2^. The composition of the purified FLiBe was measured using inductively coupled plasma-optical emission spectroscopy (ICP-OES) at the Wisconsin State Laboratory of Hygiene (WSLH). Both differential scanning calorimetry (DSC) and X-ray diffraction measurements confirmed the structure and composition of the salt. The composition of LiF and BeF_2_ was 65 ± 2 mol % and 34 ± 1 mol %, respectively. Table 1 summarizes the results of trace metal impurity measurement. The concentrations of common corrosion products such as Cr and Ni were comparable to salts produced during the MSRE^34^. Iron was below the ICP-OES detectable limit of 19 mg/kg.

Corrosion capsules were constructed of 316 L stainless steel, made with a 127 mm long tube with an inner diameter of 19 mm and wall thickness 1.65 mm. The inside of the tube was polished with Scotch-Brite General Purpose Hand Pad 7447. A polished stainless steel cap was welded with 316 L stainless steel filler rod onto the bottom of one end of the tube to form a capsule. The completed capsule was then sonicated in Oakite 33 solution at approximately 60 °C for 12 hours to remove residual machining oils and oxides. The capsules were then transferred to acetone sonication bath and then finally rinsed with ethanol and deionized water. The corrosion capsule was loaded with approximately 35.1 g of FLiBe salt, which was calculated to be enough to fully submerge the corrosion test coupons. Samples were suspended by a 316 L stainless steel wire from the 316 L top cap. The sample coupons were therefore in electrical contact with the corrosion capsule. The tube was then placed in a larger stainless steel cup which served both as a lifting mechanism as well as a containment against any possible salt leaks from the corrosion capsule^35,36^.

Samples were characterized at the Wisconsin Centers for Nanoscale Technologies with a LEO 1530 scanning electron microscopy (SEM) Energy Dispersive Spectroscopy (EDS). X-ray diffraction was performed using Bruker D8 Discover diffractometer with Cu-K_α_ micro x-ray source. The instrument has a Vantec 500 area detector with 140 mm diameter active area. The inductively coupled plasma-optical emission spectroscopy (ICP-OES) measurements were performed in the Wisconsin State Hygiene Laboratory. Mass changes were measured with a Sartorius balance with sensitivity of two micrograms.

The diffusion simulation via the CALPHAD (Calculation of Phase Diagrams) approach^37–40^ was used to further understand the experimental observations in the current study. Using the PanDiffusion module of the Pandat software^41^ and the thermodynamic + atomic mobility database developed by CompuTherm, LLC, the composition files of the sample at the diffusion affected region were simulated. The diffusion configuration sets the bulk substrate with composition of Cr_17.5_Fe_27.5_Mn_27.5_Ni_27.5_ and assigns the diffusion flux of each element at one end as a function of the experimentally measured composition Cr_25_Fe_37.5_Ni_37.5_ at the surface.

## Conclusions

The corrosion resistance of Cr_18_Mn_27_Fe_27.5_Ni_27.5_ high entropy alloy in molten salts was tested after exposure to molten FLiBe salt at 700 °C for 1000 hours. The alloy lost significant mass compared to reference 316 H stainless steel which was attributed to the dissolution of Mn into the salt. The concentration of other elements in the HEA showed no significant change - most significantly the retention of much of the Cr in the alloy rather than dissolution into the salt is an important finding of this research. Elemental distribution and bulk x-ray diffraction showed that the alloy experienced the precipitation of a Cr-rich second phase. CALPHAD calculations confirmed this result via the prediction of a new BCC phase with approximately 4% phase fraction and a nominal composition of Cr_67_Fe_13_Mn_18.5_Ni_1.5_.
